# Regulation of Hedgehog Signal Transduction by Ubiquitination and Deubiquitination

**DOI:** 10.3390/ijms222413338

**Published:** 2021-12-11

**Authors:** Qing Zhang, Jin Jiang

**Affiliations:** 1 State Key Laboratory of Pharmaceutical Biotechnology, Model Animal Research Center, School of Medicine, Nanjing University, Nanjing 210061, China; 2 MOE Key Laboratory of Model Animals for Disease Study, Model Animal Research Center, School of Medicine, Nanjing University, Nanjing 210061, China; 3 Department of Molecular Biology, UT Southwestern Medical Center, Dallas, TX 75390, USA; 4 Department of Pharmacology, UT Southwestern Medical Center, Dallas, TX 75390, USA

**Keywords:** hedgehog, Ptc, Ptch1, Smo, Ci, Gli, ubiquitination, deubiquitination, E3, DUB

## Abstract

The Hedgehog (Hh) family of secreted proteins governs embryonic development and adult tissue homeostasis in species ranging from insects to mammals. Deregulation of Hh pathway activity has been implicated in a wide range of human disorders, including congenital diseases and cancer. Hh exerts its biological influence through a conserved signaling pathway. Binding of Hh to its receptor Patched (Ptc), a twelve-span transmembrane protein, leads to activation of an atypical GPCR family protein and Hh signal transducer Smoothened (Smo), which then signals downstream to activate the latent Cubitus interruptus (Ci)/Gli family of transcription factors. Hh signal transduction is regulated by ubiquitination and deubiquitination at multiple steps along the pathway including regulation of Ptc, Smo and Ci/Gli proteins. Here we review the effect of ubiquitination and deubiquitination on the function of individual Hh pathway components, the E3 ubiquitin ligases and deubiquitinases involved, how ubiquitination and deubiquitination are regulated, and whether the underlying mechanisms are conserved from *Drosophila* to mammals.

## 1. General Introduction

First identified in *Drosophila* and later shown to be conserved in vertebrates, the Hedgehog (Hh) family of secreted proteins plays essential roles in both embryonic development and adult tissue homeostasis; not surprisingly, dysregulation of Hh signaling has been linked to many kinds of human disorders including birth defects such as holoprosencephaly and Gorlin syndrome, as well as many types of cancer including basal cell carcinomas (BCC) and medulloblastoma (MB) [1,2,3,4]. Given the critical roles that Hh signaling plays, it is not surprising that the pathway activity is tightly regulated through multiple mechanisms at different layers including the expression of Hh ligands and other pathway components as well as the perdurance, localization and activity of individual signaling components to ensure appropriate pathway activities essential for cell growth, differentiation, and pattern formation. Among these regulatory mechanisms, post-translational modifications (PTMs) including phosphorylation, ubiquitination and sumoylation are prevalent in the regulation of Hh signaling outputs [3,5,6,7,8,9,10,11]. In this review, we focus on how ubiquitination and deubiquitination regulate Hh signal transduction.

## 2. Overview of Hh Signaling Pathway

**Figure 1:**
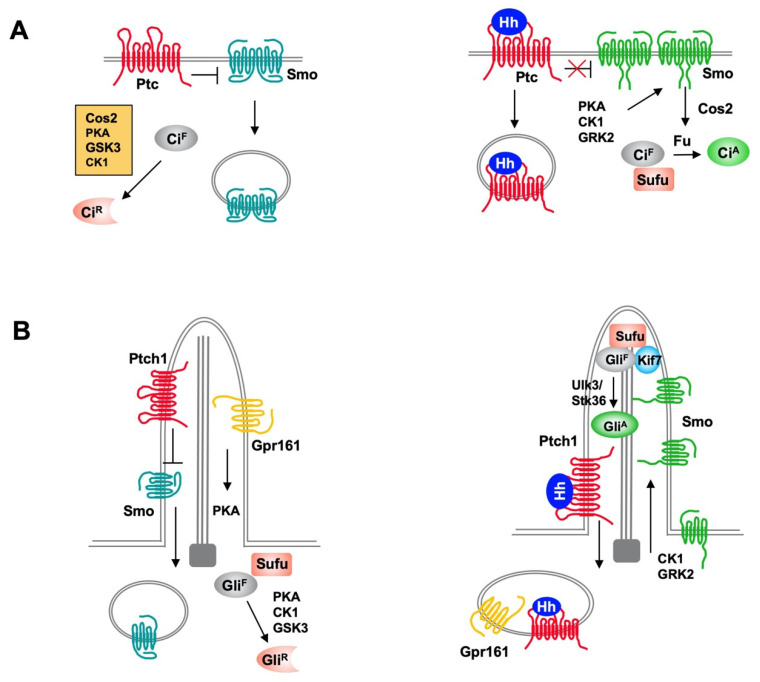
Hh signaling pathways in *Drosophila* and mammals. (**A**) In *Drosophila,* Ptc inhibits Smo activity and promotes its internalization and degradation in the absence of Hh. Full-length Ci (Ci^F^) undergoes hyperphosphorylation by PKA, GSK3, and CK1, which is facilitated by Cos2, and phosphorylated Ci is proteolytically processed to generate a truncated repressor form (Ci^R^) that inhibits Hh target gene expression. In the presence of Hh, binding of Hh inhibits Ptc activity and promotes Ptc internalization and degradation, and increases Smo cell surface accumulation and activity through PKA, CK1 and GRK2-mediated phosphorylation. Activated Smo blocks Ci^R^ production and promotes Ci^A^ formation by releasing Sufu-mediated inhibition of Ci through Fu. (**B**) In mammal, both the production of Gli repressor (Gli^R^) and activator (Gli^A^) depend on primary cilia. In the absence of Hh, ciliary localized Ptc inhibits Smo and promotes its ciliary exit and ciliary localized GPR161 activates PKA through local production of cAMP to promote Gli phosphorylation, which leads to processing of Gli^F^ to Gli^R^. In the presence of Hh, Shh inhibits Ptc and induces ciliary exit of Ptc, allowing Smo to be activated and accumulated in cilia. Smo promotes ciliary exit of GPR161 to inhibits Gli^R^ formation and promotes Gli^A^ formation through Ulk3/Stk36-mediated phosphorylation of Gli.

The Hh pathway is largely conserved from *Drosophila* to vertebrates (Figure 1) [3,12]. The core *Drosophila* Hh signal transduction pathway consists of a Hh ligand, a twelve-span transmembrane protein Patched (Ptc) that functions as the Hh receptor, a GPCR family of seven-span transmembrane protein Smoothened (Smo) that functions as the obligatory signal transducer, intracellular signaling complexes containing the kinesin-like protein Costal-2 (Cos2), the Ser/Thr kinase Fused (Fu) and the zinc finger transcription factor Cubitus interruptus (Ci) that regulates Hh target gene expression. Ci also exists in a separate complex with the Hh pathway inhibitor Suppressor of fused (Sufu). In the absence of Hh ligand, Ptc keeps the signaling pathway off by inhibiting Smo activity and preventing its accumulation on the plasma membrane. The Cos2/Fu recruits multiple kinases including protein kinase A (PKA), glycogen synthase kinase 3 (GSK3), and casein kinase 1 (CK1) to sequentially phosphorylate Ci [13]. Hyperphosphorylation targets the full-length Ci (Ci^F^) for ubiquitin/proteasome-mediated proteolysis to generate a truncated form that acts as transcriptional repressor (Ci^R^) of Hh target genes [14,15]. In the presence of Hh, binding of Hh to Ptc alleviates its inhibition of Smo [16], allowing Smo to signal downstream to block Ci^R^ production and promote Ci^F^ into an activator (Ci^A^) form [13,17].

Mammals have three Hh family proteins: Sonic hedgehog (Shh), Indian hedgehog (Ihh) and Desert hedgehog (Dhh); two Ptc proteins: Ptch1 and Ptch2; and three Gli family transcription factors: Gli1, Gli2, and Gli3, which regulate the expression of Hh target genes. The repressor (Gli^R^) and activator (Gli^A^) forms of Gli are derived mainly from Gli3 and Gli2, respectively. Gli1 is a transcription activator and direct transcriptional target of the Hh pathway. Hence, Gli1 acts in a positive feedback loop to amplify Gli^A^ activity. Vertebrate Hh signal transduction occurs at primary cilium, a microtube-based membrane protrusion found on most mammalian cells including cancer cells (Figure 1) [18,19]. Both the production of Gli repressor (Gli^R^) and activator (Gli^A^) depend on primary cilia. Hh induces reciprocal trafficking of Ptc and Smo within primary cilia with Ptc moving out of whereas Smo accumulating in cilia in response to Hh stimulation [20,21].

## 3. Ubiquitination and Deubiquitination

**Figure 2:**
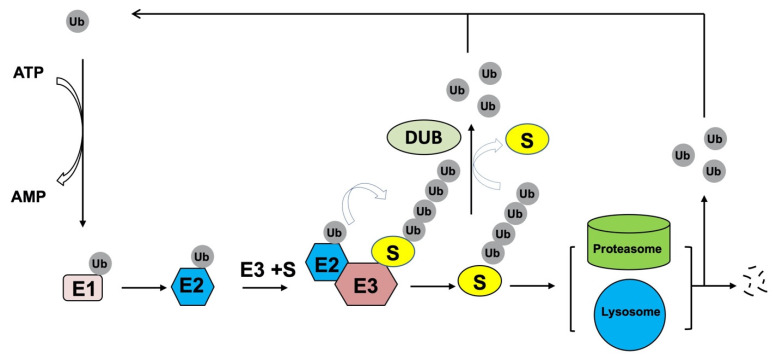
Ubiquitin pathway. During the catalytic reactions of ubiquitination, the small ubiquitin (Ub) protein is initially activated by a ubiquitin activating enzyme (E1) in an ATP-dependent manner, then transferred to the active cysteine residue of a ubiquitin conjugating enzyme (E2), and finally attached to a specific substrate through an isopeptide bond. Substrate specificity is determined by a cognate ubiquitin ligase (E3) that binds the substrate and E2. Typically, polyubiquitylation controls protein homeostasis by targeting substrates for degradation by the 26S proteasome and/or lysosome. Deubiquitinating enzymes (DUBs), which hydrolyze isopeptide or peptide bond, can remove Ub conjugates from substrates and disassembling anchored Ub chains to reverse ubiquitination process. The released ubiquitin molecules can be recycled.

Ubiquitylation is one of most prevalent PTMs which dictates the fate and function of most cellular proteins. It is crucial for a plethora of physiological processes, including cell survival, proliferation, differentiation, and innate and adaptive immunity [22]. Conducted sequentially by three different enzymes: ubiquitin-activating enzyme E1, ubiquitin-conjugating enzyme E2, and ubiquitin ligase E3, the 76 amino acids small protein ubiquitin (Ub) is finally added to specific cognate substrates to sort them to different fates (Figure 2) [23]. During the catalytic reactions, Ub is firstly activated by an E1 in an ATP-dependent manner, subsequently transferred to the active cysteine residue of an E2 via a trans-(thio) esterification reaction, and finally attached with an isopeptide bond to a substrate by an E3 that provides specificity for substrate recognition.

According to the mechanism of catalysis and their functional domains, E3s are divided into three main families: the Really Interesting New Gene (RING), the Homologous to the E6-associated protein Carboxyl-Terminus (HECT) types and RING-between-RING (RBR) [24]. The transfer of the Ub moiety to substrate is mediated through the formation of the covalent bond between α-carboxyl group of the terminal glycine residue of Ub and ε-amino group of an internal lysine (Lys) residue of the substrate. By simultaneously binding to both an E2 enzyme and a substrate, the RING family of E3s catalyzes direct transfer of ubiquitin from the E2 to the substrate. By contrast, the HECT and RBR family E3s ubiquitinate substrates in a two-step reaction in which ubiquitin is initially transferred from the E2 to an active site cysteine in the E3 and then from the E3 to the substrate. Based on different linkages of Ub on target proteins, there exist different forms of ubiquitination. Monoubiquitylation and multiubiquitylation correspond to attachment of a single Ub moiety on a single substrate residue or on multiple residues of a substrate, respectively, whereas polyubiquitylation includes attachment of additional Ub molecules to the initial Ub yielding a polyubiquitin chain [25,26]. Typically, mono- and multiubiquitylation regulate protein subcellular localization through endocytosis or protein activity to influence signal transduction. By contrast, polyubiquitylation mainly controls protein homeostasis through mediating the target substrates degradation by the 26S proteasome [26,27].

Ub harbors seven Lys (K) residues (K6, K11, K27, K29, K33, K48 and K63) that may serve as polyubiquitylation sites. Depending upon the Lys used, length of the chains and linkage types, many distinctive forms of Ub chains may be created to determine the fate of target proteins [25]. In addition to regulating proteasome-mediated protein degradation, polyubiquitylation can also regulate other cellular events depending on Ub chain linkage-types. Usually, Lys48-linkage targets protein for proteasome-dependent degradation, whereas Lys63-linkage is associated to regulation of protein localization, trafficking, protein–protein interaction; however, the biological significance of other Ub modifications is less explored [26].

Like other post-translational protein modifications, ubiquitination is a dynamic and reversible process by the action of deubiquitinating enzymes (DUBs) which hydrolyze isopeptide or peptide bond removing Ub conjugates from substrates and disassembling anchored Ub chains (Figure 2) [25]. Mechanistically, DUBs may directly interact with specific substrates or selectively recognize Ub chain architecture to cleave Ub moieties from the distal end or within chains. Through opposing E3 ligases function, DUBs control protein homeostasis and activities to regulate various physiological and pathological processes, such as development, metabolism, immune response and tumorigenesis [10].

The human genome contains about 100 DUBs which are classified into six main families according to their distinct catalytic domains: ubiquitin-specific proteases (USPs), ubiquitin C-terminal hydrolases (UCHs), ovarian tumor proteases (OTUs), Machado-Joseph disease domain proteases (MJDs or Josephins), motif interacting with Ub-containing novel DUB family (MINDYs), and JAD/PAD/MPN-domain containing metalloenzymes (JAMMs) [28]. Each family may display substrate or linkage preferences. With the exception that the JAMMs family belongs to a zinc-dependent metalloproteinases, all the other families of DUBs are cysteine proteases. To date, accumulating evidence shows both ubiquitination and deubiquitination are actively involved in the regulation of Hh signaling at multiple steps (Table 1). In addition, several ubiquitination and deubiquitination enzymes regulate ciliogenesis and thus indirectly influence Hh signaling [29,30,31,32].

## 4. Regulation of Ptc and Smo by Ubiquitination

**Figure 3:**
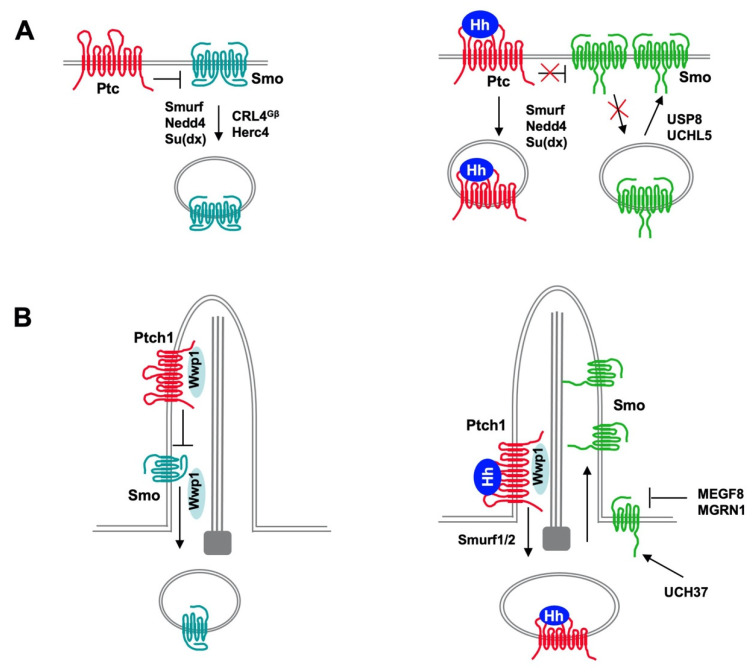
Regulation of reciprocal trafficking of Ptc and Smo by ubiquitination. In both *Drosophila* and vertebrates, Hh induces reciprocal trafficking of Ptc and Smo through regulating their ubiquitination. (**A**) In *Drosophila*, Ptc inhibits Smo in the absence of Hh, allowing Smo to be ubiquitinated by multiple E3 ligases including Smurf, Nedd4, Su(dx), CRL4^Gβ^ and Herc4, which promotes Smo internalization and degradation by both proteasome and lysosome. Binding of Hh promotes Ptc internalization and degradation mediated by E3 ligases Smurf, Nedd4 and Su(dx). Hh promotes Smo cell surface accumulation through inhibiting its ubiquitination by dissociating E3s and recruiting DUBs USP8 and UCHL5. (**B**) In mammal, Wwp1 is recruited to primary cilia through binding to Ptch1 and ubiquitinates Smo to promote its ciliary exit whereas Hh stimulates Ptch1 internalization and degradation by the E3 ligases Smurf1 and Smurf2. In addition, Smo level is inhibited by HERC4 and a membrane localized E3 complex MEGF8/MGRN1 but increased by the DUB UCH37.

The Hh signal reception system consists of two transmembrane proteins: Ptc that functions as Hh receptor and Smo that functions as Hh signal transducer [3]. In the absence of Hh ligand, Ptc actively blocks Hh signal transduction by inhibiting Smo. Binding of Hh to Ptc alleviates its inhibition of Smo, allowing Smo to transduce the signal to the intracellular compartment. In both *Drosophila* and vertebrates, Hh induces reciprocal trafficking of Ptc and Smo through regulating their ubiquitination (Figure 3).

### 4.1. Regulation of Ptc/Ptch1 by Nedd4 Family of E3s

Ptc (Ptch1 and Ptch2 in mammals) is a twelve-span membrane protein that functions as Hh receptor and is structurally related to the resistance-nodulation-division (RND) family transporters. Recent structural and functional studies revealed that Ptch1 appears to function as a sterol transporter to remove the endogenous Smo ligands, cholesterol and oxysterols away from Smo, thus keeping Smo inactive; binding of Hh to Ptch1 may block this transporter activity, allowing Smo to get access to its ligands [16,71,72,73,74,75,76,77,78,79]. Ptc/Ptch1 is a direct target of Hh signaling and its upregulation in response to Hh forms a negative feedback loop to modulate Hh signaling. The subcellular localization and stability of Ptc is also regulated by Hh. In *Drosophila*, Hh induces Ptc internalization through endocytosis and subsequent degradation whereas in mammals, Hh induces the exit of Ptch1 from primary cilia, allowing Smo to accumulate in the cilia [20,21,80].

*Drosophila* Ptc C-terminal cytoplasmic domain (CTD) mediates Ptc internalization and degradation and contains a PPXY motif that recruits multiple HECT and WW domain ubiquitin ligases including Nedd4, Smurf and Su(dx) [33,34,35]. Deletion of CTD stabilized Ptc on the cell surface while mutating the PPXY motif stabilized Ptc in intracellular vesicles or on the cell surface depending on the exact mutations made [33,34]. Mutating the PPXY motif also diminished Hh-induced Ptc internalization and degradation [34]. Although overexpression of Smurf, Nedd4, or Su(dx) could promote Ptc internalization and turnover, loss of function of individual E3s only modestly elevated Ptc in vivo [34,35], suggesting that these E3s act through partially redundant mechanisms. Consistent with this observation, overexpression of Smurf, Nedd4, or Su(dx) promoted Ptc ubiquitination; knockdown of individual E3s reduced whereas simultaneous knockdown of all three E3s abolished both the basal and Hh-induced Ptc ubiquitination in S2 cells [35,39]. K1261 in Ptc CTD was identified as a major site for Smurf-mediated ubiquitination in S2 cells and both K48-linked and K63-linked polyubiquitination chains were detected. Consistent with this, Ptc could be stabilized by both proteasome inhibitor and lysosome inhibitor and internalized Ptc colocalized with both endosome and lysosome markers [34,35]. How Hh stimulates Ptc ubiquitination and internalization is not fully understood but one study showed that Hh increased the pool of E3s accessible for Ptc by dissociating these E3s from Smo and further stimulated their binding to Ptc [39]. Indeed, knockdown of Smo increased both the basal and Hh-induced Ptc ubiquitination in S2 cells and deletion of Smo in developing *Drosophila* wings downregulated Ptc [39].

Vertebrate Ptch1 contains two PPXY motifs (one located in CTD and the other in the third intracellular loop) that mediate its internalization and degradation by recruiting Smurf/Nedd4 family of HECT-domain E3s including Smurf1, Smurf2 and Itch, all of which were shown to bind and ubiquitinate Ptch1 depending on the PPXY motifs [36,37]. Mutating the PPXY motifs or removal of Smurf1 and Smurf2 from MEF cells not only diminished Shh-induced endocytic turnover of Ptch1 but also prevented Ptch1 exit from primary cilia as well as Smo accumulating in primary cilia [36]. Consistent with these observations, removal of both Smurfs inhibited Shh target gene expression in MEFs and abolished the ability of Shh to sustain the proliferation of postnatal granule cell precursors in the cerebellum [36]. Morpholino (MO)-based knockdown of Smurf1 and Smurf2 also affected Shh signaling in zebrafish embryos. These results suggest that the Nedd4 family of E3s plays a conserved role in mediating Ptc internalization and degradation, which is stimulated by Hh.

### 4.2. Regulation of Ptch1 Ubiquitination by TSPAN8 and ATXN3

Although multiple Ptc E3s have been identified, whether DUBs are involved in reversing Ptc ubiquitination during development remains unknown. However, a recent study reported that Tetraspanin-8 (TSPAN8), a tetraspanin family member upregulated in breast cancer stem cells (CSCs) and correlated with poor prognosis in breast cancer patients, interacted with Ptch1 and inhibited its polyubiquitination and turnover by recruiting a deubiquitinase Ataxin3 (ATXN3) [38]. Intriguingly, stabilization of Ptch1 by TSPAN8 promoted Ptch1 exit from and Smo accumulation in primary cilia, resulting Hh pathway activation, which was thought to mediate the role of TSPAN8 in promoting cancer stemness and chemotherapy resistance [38]. How does TSPAN8/ATXN3-mediated deubiquitination and stabilization of Ptch1 promoted Ptch1 ciliary exit and Smo ciliary localization is perplexing; however, the authors showed that TSPAN8 promoted the binding of G protein coupled receptor kinase-2 (GRK2) to both Ptch1 and Smo. Consistent with previous findings that GRK2 binds Smo to promote its phosphorylation and activation in mammalian cells [81,82], TSPAN8 also promoted Smo phosphorylation through ATXN3 [38]. However, it is unclear how TSPAN8/ATXN3-mediated deubiquitination and stabilization of Ptch1 promoted Ptch1 ciliary exit and whether this is promoted by GRK2. It is also unclear whether TSPAN8 and/or other tetraspanin family members are involved in regulating Ptc and Hh signaling during embryonic development and adult tissue.

### 4.3. Regulation of Drosophila Smo Trafficking and Turnover by Ubiquitination

In *Drosophila*, Hh induces reciprocal trafficking of Ptc and Smo with Ptc internalized and degraded while Smo accumulated on the cell surface [80]. How Smo trafficking is regulated remained a mystery for some time but Smo phosphorylation by multiple kinases including PKA, CK1, and GRK2 promote its cell surface expression and activity [83,84,85]. Subsequent studies showed that Smo cell surface clearance is promoted by Smo ubiquitination, which is inhibited by Hh [42,43]. Genetic mutation of the sole ubiquitin activation enzyme Uba1 in developing wings (wing imaginal discs or wing discs) resulted in cell autonomous accumulation of Smo on the cell surface in the absence of Hh whereas Uba1 RNAi or pharmacological inhibition of Uba1 inhibited Smo ubiquitination and promoted Smo accumulation on the cell surface of cultured S2 cells [42]. In the absence of Hh, Smo undergoes both multi-ubiquitination and poly-ubiquitination that target Smo for both lysosome- and proteasome-mediated degradation [42,43]. Consistent with this, genetic mutations of endocytic pathway components resulted in Smo accumulation in wing discs whereas pharmacological inhibition of either lysosome or proteasome resulted in Smo stabilization in S2 cells [42,43]. Smo ubiquitination was inhibited by Hh through PKA/CK1-mediated phosphorylation of Smo C-tail, resulting in Smo cell surface accumulation [42,43]. These studies also identified USP8/UBPY as a DUB that binds and deubiquitinates Smo. Loss of USP8/UBPY blocked Hh-induced Smo accumulation whereas overexpression of USP8/UBPY resulted in ectopic Smo accumulation and Hh pathway activation [42,43]. A subsequent study revealed that Hh stimulates Smo sumoylation, which in turns recruits USP8/UBPY to promote deubiquitination of Smo, and that this process is independent of Smo phosphorylation by PKA/CK1 [6].

Although the aforementioned studies identified USP8/UBPY as a Smo DUB [42,43], these two studies failed to reveal any E3 ligases responsible for Smo ubiquitination and did not explain how Hh-induced phosphorylation of Smo by PKA/CK1 inhibited its ubiquitination. The failure of identifying a Smo E3 by genetic screen for Hh related morphological defects could be due to functional redundancy of multiple E3s involved in regulating Smo. Indeed, using a sensitive and quantitative cell-based ubiquitination assay coupled with RNAi screen targeting all the HECT domain containing E3s, Li et al. identified Nedd4 family members including Smurf, Nedd4, and Su(dx) as candidates for Smo E3s [39]. In S2 cells, Smurf RNAi reduced Smo ubiquitination and promoted Smo cell surface accumulation. Although RNAi of either Nedd4 or Su(dx) alone did not have a discernible effect on Smo ubiquitination and cell surface accumulation, their RNAi could enhance the effect of Smurf RNAi; on the other hand, overexpression of any one of these E3s promoted Smo ubiquitination and inhibited Smo cell surface accumulation [39]. Overexpression of Smurf in wing discs prevented Hh-induced Smo cell surface accumulation and blocked Hh target gene expression; however, knockdown of Smurf in wing discs only resulted in modest Hh-independent accumulation of Smo likely due to the functional redundancy with other Nedd4 family members [39].

Unlike Ptc, which recruits Smurf through its PPXY motif that binds the WW domains in Smurf, Smo recruits Smurf through the Smo autoinhibitory domain (SAID) located in the Smo C-tail, which interacts with HECT domain of Smurf [39]. The SAID domain contains multiple PKA/CK1 phosphorylation clusters essential for Hh-induced Smo phosphorylation, cell surface accumulation and activation [83,84]. Indeed, Smurf binding to Smo is inhibited by Hh through PKA/CK1-mediated phosphorylation of SAID, which explains why Smo ubiquitination is inhibited by Hh-induced Smo phosphorylation [39]. Interestingly, the interaction between Smo and Smurf is regulated by GRK2, which phosphorylates an N-terminal region of Smurf and releases an intramolecular interaction between the N-terminal region and the C-terminally located HECT domain [39]. In the absence of GRK2, the intramolecular interaction masks the HECT domain, which prevents its interaction with Smo, leading to diminished Smo ubiquitination [39]. This regulatory mechanism could explain why Smo was stabilized in *GRK2* mutant cells observed by previous studies [85]. The study by Li et al. also revealed that Smo and Ptc competes for the same pool of E3s. Hh-induced release of Nedd4 family members from Smo increased the their accessibility to Ptc, and Hh binding to Ptc further stimulated the binding of these E3s to Ptc, resulting in increased Ptc ubiquitination, internalization and degradation [39]. These regulatory mechanisms of E3 recruitment could explain why Hh induces reciprocal trafficking of Smo and Ptc [80].

### 4.4. Regulation of Mammalian Smo Ciliary Trafficking by Ubiquitination

Vertebrate Hh signaling depends on primary cilia. In response to Hh, the Hh pathway components including Smo accumulate in the primary cilia to transduce the Hh signal [20]. Previous studies suggested that ciliary trafficking of Smo is regulated by intraflagellar transport components BBSome and IFT27 [86]. Whether ubiquitination of Smo is involved in the regulation its ciliary trafficking remained unknown until recently. A recent study by Desai et al. found that Smo was ubiquitinated and removed from cilia in a manner depending on IFT27 and BBSome components [87]. Hh decreased Smo ubiquitination and ciliary exit, resulting in its ciliary accumulation. Furthermore, blockage of Smo ubiquitination by pharmacologically inhibiting E1 activity or by mutating two ubiquitin attachment sites in the third intracellular loop of Smo resulted in ectopic Smo ciliary accumulation [87].

To identify regulators of Smo ubiquitination and ciliary localization, Lv et al. carried out a candidate gene screen using CRISPR to eliminate ubiquitin pathway genes related to Hh signaling and primary cilia in engineered MEF cells expressing tagged Smo and 8XGliBS-GFP reporter, which identified several genes whose inactivation resulted in ectopic Smo ciliary accumulation, including cytoplasmic E3s (Arih2, Mgrn1, and Maea), a ciliary localized E3 (Wwp1), a ciliary localized E2 (Ube2l3), a deubiquitinase (Bap1), and three adaptors (Kctd5, Skp1a, and Skp2) [45]. The authors further showed that ciliary localization of Wwp1 depends on its interaction with Ptch1 through two its WW-domains and the two PPXY motifs in Ptch1, and Ptch1-mediated ciliary localization of Wwp1 is required for ciliary exit of Smo [45]. Cilium-targeted expression of AMSH, a DUB that selectively removed K63-linked Ub chain, resulted in ciliary accumulation of Smo, suggesting that K63-linked ubiquitin chain promotes its ciliary exit [45,46]. Consistent with this notion, an early study revealed that Wwp1 catalyzed the formation of linear K63-linked ubiquitin chain exclusively in the early phase of reaction before the formation of branched and mix-Ub linkage chains [88]. It awaits to be determined whether ubiquitination of Smo regulates its ciliary exit directly by modulating its binding to BBSome and/or other IFT proteins or indirectly by promoting its endocytosis near the cilium base, a mechanism proposed for ciliary removal of Ptch1 by Smurf1/2 [36]. It is interesting to note that Wwp1 belongs to the Nedd4 family of E3 and its closest homolog in *Drosophila* is Su(dx), followed by Nedd4 and Smurf, all of which bind both Smo and Ptc for their ubiquitination and cell surface clearance. It is intriguing that Wwp1 binds Ptch1 but does not regulate its ciliary exit while Ptch1 ciliary exit is promoted by Smurf1/2. It awaits to be determined how this specificity is determined.

### 4.5. Regulation of Smo by Other E3s and DUBs

In addition to the Nedd4 family E3s, E3s of other families have also been identified to regulate Smo ubiquitination, trafficking and turnover in *Drosophila* and mammals. In an in vivo RNAi screen for genes whose loss of function resulted in Smo accumulation, Li et al. identified a Cullin family member Cullin4 (Cul4) and its binding partner DDB1 as regulator of Smo cell surface expression in wing discs [40]. Cul4 forms multi-subunit E3 ubiquitin ligase complexes (modular RING family of E3s), in which DDB1 bridges Cul4 to multiple DDB1-binding WD40 (DWD) proteins that recognize the specific substrates [89]. Further studies revealed that Smo C-tail recruits Cul4-DDB1 through multiple β subunits of trimeric G proteins (Gβs), which are DWD proteins, and that Cul4-DDB1-Gβ promotes the Smo ubiquitylation and turnover. Hh signaling disrupts the interaction between DDB1 and Gβ by recruiting the catalytic subunit of PKA (PKAc) to phosphorylate DDB1, which dissociates Cul4-DDB1 from Smo and results in elevated Smo cell surface expression [40].

A genetic modifier screen for genes whose RNAi could modify the wing phenotype caused by overexpression of a dominant negative Smo identified UCHL5 (thiol protease class of DUB whose mammalian counterpart is UCH37) and Herc4 (HERC family E3) that contains a HECT domain and RCC1-like domains (RLDs) as regulators of Smo ubiquitination and cell surface expression [41,44]. UCHL5 interacts with Smo to promote its deubiquitination and accumulation at the cell membrane, and the interaction between UCHL5 and Smo is enhanced by Hh [44]. Furthermore, knockdown of UCH37 in NIH3T3 cells reduced human Smo level and Shh pathway activity, suggesting that UCHL5/UCH37 plays a conserved role in modulating Smo ubiquitination and turnover [44]. Herc4 interacts with Smo SAID domain through both its HECT and RCC1-like domains to mediate Smo ubiquitination and degradation [41]. Hh inhibits Herc4-mediated Smo ubiquitination by two distinct mechanisms: one involves recruiting UCHL5 to compete out Herc4 binding and the other involves PKA/CK1 phosphorylation-induced conformational change of Smo C-tail that masks the ubiquitin acceptor sites [41]. Another study showed that mammalian HERC4 acts as a tumor suppressor via destabilizing the oncoprotein Smo [47]. In non-small cell lung cancer (NSCLC) samples, HERC4 is downregulated and negatively correlated with Smo. HERC4 interacted with Smo in NSCLC cells and knockdown of HERC4 activated Hh pathway and promoted NSCLC cell proliferation [47]. Taken together, these studies identified a conserved pair E3 and DUB that modulate Smo ubiquitination and turnover.

In a genome-wide screen for attenuators of Hh signaling, MEGF8, a transmembrane protein, and MGRN1, a RING superfamily E3 ligase were identified whose loss-of-function resulted in an elevated response to Shh ligands due to the accumulation of Smo at the cell surface and primary cilium [90]. Further biochemical study showed that MGRN1 and MEGF8 formed a receptor-like E3 ligase complex to promote the ubiquitination and degradation of Smo. Homozygous Megf8 and Mgrn1 mutations increased Smo abundance and elevated sensitivity to Hh ligands [90]. Mice heterozygous for loss-of-function Megf8 or Mgrn1 mutations were normal but double heterozygous embryos exhibited an incompletely penetrant syndrome of congenital heart defects (CHDs), indicating that fine genetic interactions between Megf8 and Mgrn1 may affect Hh signaling strength through mediating Smo degradation, which contributes to CHDs [90].

## 5. Regulation of Hh Pathway Transcription Factors Ci/Gli by Ubiquitination and Deubiquitination

**Figure 4:**
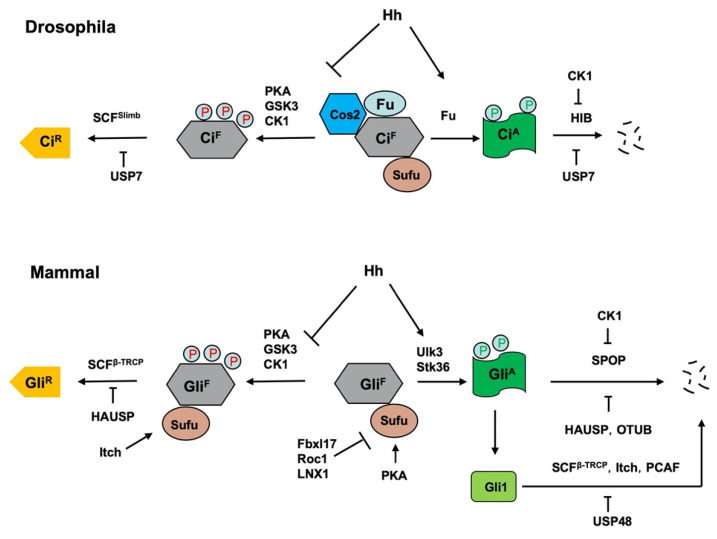
Regulation of Ci/Gli processing and degradation by E3s and DUBs. Hh signaling controls the balance between the repressor and activator forms of the Ci/Gli, which is regulated by the ubiquitin and proteasome system (UPS). In the absence of Hh, full-length Ci/Gli (mainly Gli3) is proteolytically processed to form truncated repressor (Ci^R^/Gli^R^) through PKA/GSK3/CK1-mediated phosphorylation, followed by SCF^Slimb^/SCF^β-TRCP^-mediated ubiquitination. The DUB USP7/HAUSP antagonizes this process through deubiquitinating Ci/Gli. The E3 ligase Itch promotes Gli^R^ production by ubiquitinating Sufu to increase its interaction with Gli3. Hh signaling blocks the proteolysis of Ci/Gli and converts accumulated full-length Ci/Gli into activator form Ci^A^/Gli^A^ though phosphorylation by the Fu family kinases. When dissociated from Sufu, Ci^A^/Gli^A^ becomes labile and is degraded by HIB/SPOP, a process that is attenuated by CK1-meidated phosphorylation of Ci/Gli. The DUBs USP7/HAUSP and OTUB deubiquitinate Ci/Gli to inhibit its degradation. The E3 ligases SCF^β-TRCP^, Itch and PCAF promote Gli1 degradation while the DUB USP48 antagonizes this process. In addition, mammalian Sufu is degraded by multiple E3 ligases including Fbxl17, ROC1 and LNX1.

Hh signaling controls the balance between the repressor and activator forms of the Ci/Gli family of transcription factor. In the absence of Hh, full-length Ci/Gli is proteolytically processed to form truncated repressor (Ci^R^/Gli^R^) that lacks the C-terminal coactivator domain but retains the N-terminal corepressor domain. Hh signaling blocks the proteolysis of Ci/Gli and converts accumulated full-length Ci/Gli into activator form Ci^A^/Gli^A^. Both the production of Ci^R^/Gli^R^ and abundance of Ci^A^/Gli^A^ are regulated by the ubiquitin and proteasome system (UPS) (Figure 4) [8,91].

### 5.1. Regulation of the Production of Ci^R^/Gli^R^ by Slimb/β-TRCP

Genetic studies in *Drosophila* identified several kinases including PKA, GSK3, and CK1 and an F-box protein Slimb (β-TRCP1 and β-TRCP2 in mammals) as essential for Ci^R^ formation [14,92,93,94,95]. Slimb serves as a substrate recognition subunit of a modular RING E3 ligase complex that contain Cul1, Skp1, and an F-box protein (SCF^Slimb^), and SCF family E3s usually bind substrates only after they are phosphorylated and thus link kinase regulation to UPS mediated proteolysis. Indeed, subsequent biochemical studies showed that PKA, GSK3 and CK1 sequentially phosphorylate Ci at three Ser/Thr clusters in its C-terminal region, which creates a docking site (or degron) for SCF^Slimb^; binding of SCF^Slimb^ to Ci then catalyzes Ci ubiquitination, which targets it for proteosome-mediated proteolysis to generate Ci^R^ [14,15,48]. The same mechanism is operating in vertebrates where PKA, GSK3 and CK1 sequentially phosphorylate the C-terminal region of Gli2 and Gli3 to create docking sites for SCF^β-TRCP^ [96], which targets Gli2/3 for ubiquitin/proteosome-mediated proteolytic processing to generate Gli^R^. A PDD (processing determinant domain) domain of Ci/Gli located between the Zn-finger DNA binding and Slimb/β-TRCP binding domains seems to be critical for proteasome-mediated removal of its C-terminal half [97]. Deletion of PDD blocks Ci^R^ formation and renders complete degradation of Ci by SCF^Slimb^-mediated ubiquitination [48]. Consistent with the observation that the Gli^R^ function is mainly contributed by Gli3 while Gli2 mainly contributes to the Gli^A^ activity, Gli3 is processed more effectively than Gli2 because Gli3 has a more potent PDD [98].

A subsequent study identified Ter94 ATPase and its mammalian counterpart, p97, as essential for Ci/Gli3 proteolytic processing into Ci^R^/Gli3^R^ [99]. Ter94 regulates the partial degradation of ubiquitinated Ci by SCF^Slimb^ through its adaptors Ufd1-like and dNpl4. This study showed that SCF^Slimb^ catalyzed K11-linked ubiquitin chain formation on Ci and Ter94/Ufd1-like/dNpl4 complex interacts with both SCF^Slimb^ and K11-linked ubiquitinated Ci to modulate partial degradation of Ci by proteasomes [99].

Hh inhibits SCF^Slimb/β-TRCP^-mediated Ci/Gli ubiquitination and proteolytic processing by inhibiting Ci/Gli phosphorylation and thereby E3 recruitment. In *Drosophila*, Ci phosphorylation is regulated by an intracellular signaling complex consisting of Cos2 and Fu that functions as a molecular scaffold to bring Ci and its kinases, PKA, GSK3 and CK1 in close proximity and thus facilitating Ci phosphorylation [13]. In response to Hh, Smo adopts an active conformation and interacts with Cos2 through its C-tail to cause dissociation of the Ci-Cos2/Fu-kinase complex [13,84,100,101,102]. In addition, Hh induces the formation of Smo-PKAc complex, which competes PKAc away from Ci [103,104]. Consequently, Ci phosphorylation by PKA is inhibited, which blocks SCF^Slimb^ recruitment. The production of Gli^R^ depends on primary cilia where local production of cAMP mediated by GPR161, a Gαs coupled GPCR located at the primary cilia in the absence of Hh, activates PKA to promote Gli phosphorylation [105]. Shh stimulates ciliary exit of Gpr161, which is thought to reduce localization concentration of cAMP and hence the PKA activity [105,106]. Furthermore, a recent study showed that Shh stimulates Smo-PKAc complex formation to sequester PKAc away from Gli in cultured mammalian cells [82], suggesting that Smo-mediated sequestration of PKAc away from Ci/Gli could be a conserved mechanism for inhibiting Ci/Gli phosphorylation, ubiquitination, and processing.

### 5.2. Regulation of Ci/Gli Degradation by HIB/SPOP

Hh signaling not only blocks Ci/Gli processing to generate Ci^R^/Gli^R^ but also converts accumulated full-length Ci/Gli (Ci^F^/Gli^F^) into its activator form Ci^A^/Gli^A^. In *Drosophila*, the Ser/Thr kinase Fu converts Ci^F^ into labile Ci^A^ by antagonizing Sufu in response to high levels of Hh [107]. In the absence of Hh, Sufu binds Ci through the N- and C-terminal region of Ci to inhibit Ci nuclear localization and the recruitment of coactivator CBP [108]. Hh stimulates Ci phosphorylation by Fu, which alters its binding to Sufu and thus converts Ci into Ci^A^ [17]. The inhibition of Sufu-Ci interaction makes Ci more accessible to HIB (Hh-induced MATH and BTB domain containing protein; also called Roadkill or Rdx) that functions as a substrate recognition subunit for the Cul3-based modular E3 ubiquitin ligase complexes [49,50,109,110]. Unlike SCF^Slimb^-mediated ubiquitination that targets Ci for partial degradation, Cul3-HIB mediated ubiquitination targets Ci for complete degradation [49]. Consistent with this, Cul3-HIB does not interact with Ter94, an ATPase that promotes partial degradation of Ci by proteasomes [99]. HIB is induced by Hh in both embryos and wing discs thus forming a negative feedback loop to attenuate Hh signaling outputs [49,50]. Inactivation of HIB increased Ci^A^ levels but did not completely block Ci^A^ turnover in wing disc cells receiving highest dose of Hh [49], and a recent study showed that CRISPR-mediated mutation of the major HIB binding sites on endogenous Ci also failed to block Ci degradation [111], implying that additional mechanism may be involved in degrading Ci^A^.

The mammalian homolog of HIB is SPOP, which plays a conserved role in promoting the degradation of full-length Gli proteins [49,53,54,55,56,57,58,59]. As in the *Drosophila*, loss of Sufu renders Gli proteins more accessible to SPOP-mediated degradation, which explains at least in part the positive role Sufu plays in the Hh pathway [53]. Loss of Sufu alone was unable to induce MB formation in mice due to SPOP-mediated degradation of Gli2; however, simultaneous loss of SPOP and Sufu restored Gli2 activity and induced rapid MB formation [59]. Similarly, simultaneous KO of Sufu and SPOP in mouse intestines caused more dramatic phenotypes associated with ectopic Hh signaling than Sufu KO alone [58]. Loss of SPOP inhibited Ihh signaling and skeletal development due to increased Gli3^R^ activity [56], which is consistent with a previous finding that both the repressor and activator forms of Ci were degraded by HIB in *Drosophila* [49].

HIB/SPOP recognizes multiple Ser/Thr (S/T)-rich motifs located in both the N- and C-terminal regions of Ci/Gli proteins [112,113], which could explain why both the truncated repressor and full-length activator forms of Ci/Gli are recognized by HIB/SPOP [49,55,56]. Recently, similar Ser/Thr motifs have been identified in other SPOP substrates including ERG oncoprotein and BET family proteins [114,115,116]. Cooperative binding to two HIB/SPOP recognition motifs present either in cis or in trans on substrates appears to be essential for HIB/SPOP-mediated ubiquitination and degradation of the substrates [112,113], suggesting that HIB/SPOP may contact its substrates through multivalent interactions. Indeed, a recent study revealed that SPOP could form a liquid–liquid phase separation (LLPS) through its BTB and BACK domain-mediated oligomerization, which may facilitate the interaction between E3s and substrates [117].

The binding of HIB/SPOP to Ci/Gli is opposed by Sufu [49,53], which binds Ci/Gli tightly through multiple contact sites, thus masking the HIB/SPOP binding sites [108,118]. In *Drosophila*, high levels of Hh downregulate Sufu through upregulation of HIB to modulate pathway activity [119]. The binding of HIB/SPOP to Ci/Gli is also inhibited by CK1 that phosphorylates multiple HIB/SPOP binding sites in Ci/Gli to block HIB/SPOP binding, which provides a mechanism to sustains Hh signaling by preventing premature loss of Ci^A^ [120]. Phosphorylation also regulates SPOP binding to a number of substrates including MacroH2A, Puckered and Pdx1, suggesting a more general mechanism for HIB/SPOP regulation [113,121].

### 5.3. Proteolytic and Non-Proteolytic Regulation of Gli by Other E3s

Unlike Gli2 and Gli3 whose ubiquitination by SCF^β-TRCP^ targets them for partial degradation, SCF^β-TRCP^-mediated ubiquitination of Gli1 targets it for complete degradation through a β-TRCP degron located in the C-terminal region of Gli1 [52]. Several recent studies suggest that β-TRCP/Gli1 interaction can be regulated by a variety of mechanisms. One study showed that high mobility group box protein SOX9 is a transcriptional target of Gli1 in KRAS-driven pancreatic ductal adenocarcinoma (PDA) and acts in a positive feedback loop to stabilize Gli1 by binding and sequestrating β-TRCP away from Gli1, which is thought to promote PDA [122]. Another group found that AMPK could directly phosphorylate Gli1 at multiple sites to promote β-TRCP binding, Gli1 ubiquitination and degradation, which is thought to modulate Hh signaling activity in MB [123,124]. Kim et al. showed that the tumor suppressor RUNX3 interacted with Gli1 to promote β-TRCP recruitment, Gli1 ubiquitination and degradation, thus inhibiting Hh signaling to suppress metastasis and stemness in colorectal cancer [125]. Park et al. reported that hypoxia stimulated the binding of T-complex protein 1 subunit beta (CCT2) to Gli1 to prevent its ubiquitination and degradation by β-TRCP in colorectal cancer cells [126]. Finally, a recent study showed that the short isoform of PHD finger protein 19 (PHF19) interacts with β-TRCP to inhibit Gli1 ubiquitination and thus promoting Hh signaling in hepatocellular carcinoma (HCC) [127].

β-TRCP is unlikely the only E3 that promotes Gli1 ubiquitination and degradation because mutating the β-TRCP dependent degron in Gli1 failed to fully stabilize Gli1 [52]. Di Marcotullio et al. identified Numb as a negative regulator of Hh signaling that was downregulated in cerebellar granule cell progenitors (GCPs) and MB. The study further showed that Numb promotes ubiquitination and degradation of Gli1 through the HECT domain E3 ligase Itch and that Numb/Itch-mediated downregulation of Gli1 inhibits Hh signaling to arrest GCP proliferation and promote its differentiation, a mechanism that could be deregulated in MB [60]. A subsequent study by the same group showed that Numb activates the catalytic activity of Itch by binding its WW domains and alleviates an inhibitory intramolecular interaction between its HECT and WW domains [128]. On the other hand, Numb also recruits Gli1 through direct binding to facilitate the binding of Gli1 to Itch through of novel Itch-dependent degron composed of a combination of two PPXYs and a phospho-serine/proline motifs located in the C-terminal region of Gli1, leading to ubiquitination and degradation of Gli1 [128]. A recent study showed that lithium, a GSK3 inhibitor, upregulated the expression of Itch to downregulate Gli1 protein levels In pancreatic ductal adenocarcinoma (PDA) cell lines; however, the mechanism by lithium promotes itch expression remains unexplored [129].

Another study identified the acetyltransferase p300/CBP-associated factor PCAF as an E3 ubiquitin ligase of Gli1 [62]. This study showed that genotoxic stress can trigger P53 dependent upregulation of PCAF, which targets Gli1 for ubiquitination and proteasome-mediated degradation, leading to inhibition of Gli activity. Restoring Gli1 levels rescued the growth arrest and apoptosis triggered by genotoxic stress and DNA-damaging agents failed to inhibit Gli1 activity in the absence of either p53 or PCAF. MB samples from p53-null mice displayed low levels of PCAF and upregulation of Gli1. Together, this study defined a mechanism of inactivation of Hh signaling in response to genotoxic stress and unveiled a p53/PCAF/Gli1 signaling axis that limits Gli1-enhanced mitogenic and prosurvival response [62].

A recent study identified WWP2, a HETC domain containing E3, as a negative regulator of Hh signaling by targeting Gli2 for ubiquitination [63]. The study further showed that WWP2 is a direct target of Wnt/β-catenin signaling pathway, and that DKK1 suppresses the expression of WWP2 by blocking the canonical Wnt signaling to promote Gli stabilization and Hh pathway activation, which may contribute to DKK1-mediated drug resistance in bortezomib-based chemotherapy of multiple myeloma [63].

In addition to regulating Gli degradation by multiple E3s, a recent study revealed that that RNF220, a RING family ubiquitin E3 ligase, regulates Hh signaling through non-degradation mechanism in neural tube patterning [61]. Mechanistically, RNF220 interacts with all Gli family members either in their activator or repressor forms to induce their K63-linked ubiquitination and promotes their nuclear export, likely by unmasking a nuclear export signal in the zinc finger domain [61]. Hence, RNF220 modulates the Gli activator and repressor gradients during neural patterning by limiting the effective Gli levels in the nucleus.

### 5.4. Regulation of Ci/Gli Processing and Degradation by DUBs

In addition to multiple E3s, several DUBs have been implicated in the regulation of Ci/Gli. Zhou, et al. showed that Hh stimulates the binding of a ubiquitin-specific protease USP7 to Ci, which positively regulates Hh signaling activity through inhibiting Ci ubiquitination and degradation mediated by both Cul1-Slimb and Cul3-HIB E3 ligases [51]. Importantly, they showed that the mammalian counterpart of USP7, HAUSP, plays a conserved role to positively regulate Hh signaling by modulating Gli ubiquitination and stability [51]. High USP7/HAUSP expression predicted unfavorable prognosis of cancer patients, especially those with epithelial ovarian cancer (EOC), suggesting that USP7/HAUSP is a critical regulator of Hh signaling and potential therapeutic target for Hh-related cancers.

Another study identified the deubiquitinating enzyme OTUB2 as a positive regulator of Gli2 protein and Hh signaling in osteogenic differentiation [64]. The authors found that OTUB2 formed a complex with Gli2 and promoted Gli2 deubiquitination both in vitro and in vivo. OTUB2 knockdown suppressed the alkaline phosphatase activity and the expression of the common osteogenic markers BMP2 and RUNX2 during osteogenesis of mesenchymal stem cells in response to Shh and Smo agonists, suggesting that OTUB2 may play a role in osteogenic differentiation by regulating Hh signaling [64].

In addition to USP7 and OTUB2, the deubiquitinase USP48 was found to activate Hh signaling by stabilizing Gli1 protein in glioma cells [65]. USP48 interacts with Gli1 through its C-terminal DUSP domain to remove ubiquitin moiety from Gli1. Knockdown of USP48 inhibited the expression of Gli1 target genes and repressed cell proliferation and tumorigenesis [65]. Interestingly, USP48 is transcriptionally activated by Gli1 in glioma cells, thus forms a positive feedback loop to regulate Hh signaling [65]. In human glioblastoma, USP48 and Gli1 expression levels were positively correlated, and high USP48 expression levels correlated with higher grades of glioma malignancy [65], suggesting that the USP48-Gli1 regulatory axis is critical for glioma cell proliferation and glioblastoma tumorigenesis.

## 6. Regulation of Hh Intracellular Signaling Components by Ubiquitination

The intracellular Hh signal transduction pathway contains several conserved signaling components including the kinesin-like protein Cos2/Kif7, the Fu family kinases Fu/Ulk3/Stk36, and the tumor suppressor Sufu [3]. In *Drosophila*, Cos2 and Fu form a complex to promote Ci phosphorylation, ubiquitination and proteolytic processing to form Ci^R^ in the absence of Hh [13]. In response to Hh stimulation, Cos2 recruits Fu to activated Smo to induce Fu dimerization/oligomerization, autophosphorylation and activation [102,130]; activated Fu then phosphorylates Ci to convert it into Ci^A^ by altering its binding to Sufu [17]. In mammalian cells, Kif7 also plays a dual role in regulating the formation of both Gli^R^ and Gli^A^ [131,132,133]; however, the underlying mechanisms remain obscured although Kif7 has been shown to organize the cilium tip compartment where Gli is thought to be activated [134,135]. The Fu family kinases Ulk3 and Stk36 play a partially redundant role in phosphorylating and activating Gli proteins by alleviating Sufu-mediated repression [17]. Several studies revealed that both Cos2/Kif7 and Sufu are regulated by the UPS.

### 6.1. Regulation of Cos2/Kif7 by UBR3

An early study revealed that Cos2 is downregulated in response to Hh in wing imaginal discs [136]; however, how Cos2 is downregulated by the UPS has remained obscured for some time. A recent study by Li et al. identified UBR3, a RING-like E3 ubiquitin ligase, as a positive regulator of Hh signaling in both *Drosophila* and vertebrates [66]. In *Drosophila* eye imaginal discs, loss of UBR3 resulted in increased Cos2 level, reduced Hh signaling activity and delayed photoreceptor differentiation. In *Drosophila* S2 cells, UBR3 binds to the N-terminal MD of Cos2 with its UBR domain and promotes Cos2 K48-linked poly-ubiquitination and degradation by proteasome, and Hh stimulated the binding of UBR3 to Cos2 to promote Cos2 ubiquitination. However, loss of UBR3 in wing discs did not completely block the Hh-induced Cos2 degradation, implying additional mechanism(s) could be involved [66]. Interestingly, *ubr3* is transcriptionally activated by Hh in eye imaginal discs, thus forming a positive loop to promote further activation of Hh signaling [66]. In zebrafish, loss of UBR3 also attenuated Shh signaling in the developing eyes, somites, and sensory neurons, but not in all tissues that require Hh signaling. In mammalian cells, UBR3 poly-ubiquitinated Kif7 to promote Hh signaling. Together, this study identified Ubr3 as a novel, evolutionarily conserved modulator of Hh signaling that boosts Hh pathway activity in certain tissues. It remains to be determined whether Cos2/Kif7 is regulated by another E3(s) in tissues where Cos2/Kif7 levels are not affected by loss of UBR3.

### 6.2. Regulation of Sufu by Multiple E3s

Sufu is a conserved inhibitory component of the Hh signaling pathway. In *Drosophila*, Hh signaling promotes downregulation of Sufu through its target gene *hib*; however, although HIB-mediated downregulation of Sufu depends on the E3 ubiquitin ligase Cul3, HIB does not directly regulate Sufu protein stability but instead, indirectly modulates cytosolic mRNA translation of Sufu, leading to reduced Sufu protein level [119]. In mammalian cells, Shh signaling promoted Sufu K48-linked poly-ubiquitination on K257 and degradation by inhibiting PKA/GSK3-mediated phosphorylation of Sufu at Ser342/S346 that normally stabilizes Sufu [137,138]. Although overexpression of stabilized Sufu variants inhibited Hh signaling more potently than wild type Sufu [137], to what extent this Hh-induced Sufu turnover contributes to Hh pathway activation under physiological conditions remains to be determined.

While these studies clearly demonstrated that Sufu turnover can be regulated by UPS, the relevant E3s remained obscured. A recent study by Raducu et al. identified Fbxl17 (F-box and leucine-rich repeat protein 17) as a bona fide E3 ubiquitin ligase for Sufu [67]. Fbxl17 formed a complex with Sufu-Gli1 in the nucleus to promote Sufu ubiquitination and degradation, leading to the release of Sufu-mediated inhibition of Gli1. Like the F-box protein Slimb/β-TRCP, Fbxl17 forms an SCF E3 ubiquitin complex with Skp1 and Cul1 to target substrates for ubiquitination. However, in contrast to Slimb/β-TRCP whose binding to substrates is promoted by substrate phosphorylation, the binding of Fbxl17 to Sufu is inhibited by PKA/GSK3-mediated phosphorylation of Sufu at Ser342/S346 [67]. Fbxl17 mRNA was found to be significantly increased in the SHH-subtype MB and the levels of Fbxl17 and Gli1 positively correlated; depletion of Fbxl17 reduced Hh signaling through stabling Sufu, attenuated cancer cell proliferation and MB tumor growth [67]. Furthermore, this study identifies S352F mutation of Sufu, occurring in MB of patients with Gorlin syndrome, destabilizes Sufu through enhanced binding to Fbxl17 [67], highlighting the perturbation of the Fbxl17-Sufu axis in the pathogenesis of MB.

Consistent with the involvement of an SCF E3 ubiquitin ligase complex in the regulation of Sufu, a recent study by Wang et al. showed that ROC1/RBX1, the RING component of cullin-based E3 ubiquitin ligase implicated in in bladder cancer cell survival and progression, could target Sufu for ubiquitination and degradation, leading to dissociation of Gli2 from Sufu and activation of Hh pathway in bladder cancer cells [69]. ROC1 levels and Sufu levels are reversely correlated in bladder cancer samples and high ROC1 levels correlated with high grade cancers. ROC1 overexpression promoted whereas knockdown inhibited the growth of bladder cancer cells. These results suggest that ROC1 may promote bladder cancer progression by targeting Sufu for degradation. However, it is unclear whether ROC1 targets Sufu for ubiquitination through Fbxl7. It is also unclear why ROC1 selectively targets Sufu but not Gli in the Hh pathway since Gli proteins are also regulated by Cullin-based E3s such as β-TRCP and SPOP.

Another study by Infante et al. identified the HECT E3 ligase Itch as an E3 ligase for Sufu [68]. Interestingly, Itch forms a complex with β-arrestin2 to bind Sufu and promote its K63-linked polyubiquitylation that does not affect Sufu stability but instead, increases the interaction between Sufu and Gli3 to promote the conversion of Gli3 into Gli3^R^, and this process is inhibited by Hh signaling [68]. Importantly, several Sufu variants from MB patients escaped from Itch-mediated regulation, leading to diminished Gli3^R^ and thus aberrant Hh signaling activity and enhanced tumor growth [68].

Finally, a recent study identified a Sufu Negating Protein 1 (SNEP1) that positively regulates Hh signaling by promoting Sufu degradation [70]. SNEP1 is a transcriptional target of Gli in colorectal cancer (CRC) cell lines and can partner with LNX1, a Ring E3 ligase, to ubiquitinate and degrade Sufu. Overexpression of SNEP1 promoted CRC cell proliferation in vitro and tumor growth in vivo. High levels of SNEP1 were detected in CRC patient samples and correlated with poor prognosis in CRC patients. Altogether, this study reveals that SNEP1 may act as a feedback regulator of Hh signaling by destabilizing Sufu to promote CRC growth.

## 7. Conclusions

Like all other developmental signaling pathways, Hh signaling is under strict regulation in development and adulthood to avoid developmental errors and malignancies. Ubiquitination- and deubiquitination-mediated proteolysis plays a particularly important role in the regulation of this pathway. Despite intensive investigations in the past two decades that lead to the identification of many E3s and DUBs involved in regulation of Hh signaling, the physiological roles of many of these regulations have remained unclear or not been fully explored. For example, ciliary exit of Ptch1 might not be absolutely required for Hh pathway activation but rather could modulate the amplitude or perdurance of pathway activity because one study showed that Hh could elicit pathway activity in cells expressing Ptch1 variants that failed to exit primary cilia [139]. Similarly, although Sufu is ubiquitinated and downregulated in Ptch1 mutant cells, whether and to what extent Sufu downregulation is essential for Hh pathway activation during development remain unclear. The involvement of multiple E3s that function partial- redundantly in the regulation of Ptc/Ptch1 and Smo makes it challenging to dissect the role of individual E3s in Hh signaling. A related question is how the specificity or selectivity of E3s is achieved. For example, multiple Nedd4 family members are involved in the regulation of *Drosophila* Smo whereas only Wwp1 but no other Nedd4 family members are involved in ciliary trafficking of mammalian Smo. Other outstanding questions include why binding of Wwp1 to Ptch1 does not promote Ptch1 ubiquitination and ciliary exit, how Ptch1 ubiquitination and ciliary exit are stimulated by Hh, and why Slimb/β-TRCP-mediated ubiquitination promotes Ci/Gli processing whereas HIB/SPOP-mediated ubiquitination promotes complete degradation.

Because deregulation of Hh signaling has been implicated in many types of cancer [3], targeting the Hh pathway has become an attractive strategy for cancer therapy. The identification of multiple E3s and DUBs that participate in the regulation of Hh signaling and the elucidation of their roles in cancer have opened the door for developing novel therapeutical strategy to treat Hh-related cancer. In this regard, several E3 ligases and DUBs are emerging as interesting therapeutic targets in various Hh-related tumors given their positive roles in Hh signaling [10]. However, it remains challenging to develop more efficient and selective inhibitors of E3 ligases and DUBs for anti-cancer therapy without affecting normal physiology.

## Figures and Tables

**Table 1:** E3 ligases and DUBs involved in Hh pathway regulation.

Hh Pathway Components	E3s	DUBs
Ptc (*Drosophila*)	Nedd4 [33,34], Smurf [34,35], Su(dx) [34]	
Ptch1 (Mammal)	Smurf1&2 [36], Itch [36,37]	ATXN3 [38]
Smo (*Drosophila*)	Smurf [39], Nedd4 [39], Su(dx) [39], CRL4^Gβ^ [40], Herc4 [41]	USP8 [42,43], UCHL5 [44]
Smo (Mammals)	Wwp1 [45], MGRN1 [45,46], HERC4 [47]	UCH37 [44]
Ci	SCF^Slimb^ [14,15,48], HIB [49,50]	USP7 [51]
Gli	SCF^β-TRCP^ [52], SPOP [49,53,54,55,56,57,58,59], Itch [60], RNF220 [61], PCAF [62], WWP2 [63]	HAUSP [51] OTUB2 [64] USP48 [65]
Cos2/Kif7	UBR3 [66]	
Sufu (Mammal)	Fbxl17 [67], Itch [68], ROC1 [69], LNX1 [70]	

## Data Availability

The data that support the findings of this study are available from the corresponding author upon reasonable request.
